# Reality check for transposon enhancers

**DOI:** 10.7554/eLife.47900

**Published:** 2019-05-31

**Authors:** Julie Brind'Amour, Dixie L Mager

**Affiliations:** 1 Department of Medical GeneticsUniversity of British ColumbiaVancouverCanada; 2 Terry Fox LaboratoryBC CancerVancouverCanada

**Keywords:** transposable elements, enhancers, embryonic stem cells, trophoblast stem cells, CRISPR, endogenous retrovirus, Mouse

## Abstract

Hundreds of retrovirus-like sequences have features that suggest they might be gene enhancers, but only a small fraction displays gene-regulating activity in experiments on mouse stem cells.

**Related research article** Todd CD, Deniz Ö, Taylor D, Branco MR. 2019. Functional evaluation of transposable elements as enhancers in mouse embryonic and trophoblast stem cells. *eLife* **8**:e44344. doi: 10.7554/eLife.44344

First described over forty years ago, an enhancer is a genetic sequence that can 'switch on' a far away gene in certain tissues or at key points during development by interacting with the promoter for that gene. While promoters are generally conserved between organisms, enhancers are often unique to a given species, suggesting that they have evolved more recently (reviewed in Long et al., 2016).

One source of species-specific enhancers might be transposable elements, DNA sequences that can copy themselves and jump to another location in the genome (or simply move to another place). Many of these elements are derived from retroviruses whose genetic code has permanently colonized the genome of their hosts (also known as endogenous retrovirus-like elements, or ERVs). In humans and mice, over 40% of chromosomal DNA is made of transposable elements. Although the vast majority are no longer capable of jumping, they are responsible for much of the genomic diversity across species.

To successfully spread through the genome, these sequences contain their own regulatory components, including enhancers and promoters. Whether cells have then ‘domesticated’ transposable elements for their own advantage – and in particular, whether certain sequences can act as dispersed ‘controlling elements’ in regulatory gene networks – has been a topic of interest for half a century (Britten and Davidson, 1969; Chuong et al., 2017), with this concept gaining momentum in the last decade.

Indeed, genome-wide studies have revealed that transposable elements can show traits associated with enhancers, such as being able to bind to transcription factors or displaying characteristic epigenetic and chromatin features (Kunarso et al., 2010; Chuong et al., 2013). These discoveries have fuelled models in which transposable elements are being co-opted to act as enhancers.

Enhancer-like epigenetic features and binding sites for transcription factors are particularly common in regions of ERVs called long terminal repeats. Still, the evidence which shows that these elements have enhancer activity remains provocative. As with any putative enhancer, the challenge is now to go beyond analyses which demonstrate correlations and towards studies that rigorously validate that transposable elements can work as enhancers (as discussed in Chuong et al., 2017). Now, in eLife, Miguel Branco and colleagues at Queen Mary University of London – including Christopher Todd as first author – report that such assessment is, indeed, critically needed (Todd et al., 2019).

The team examined families of ERVs whose long terminal repeats can bind to transcription factors and which show the classic epigenetic features associated with enhancers, such as open chromatin and certain histone modifications (Figure 1A). In particular, they focused on elements that had been reported to contain binding sites for key transcription factors which are specific to mouse embryonic or trophoblast stem cells (Kunarso et al., 2010; Chuong et al., 2013; Sundaram et al., 2017). This allowed Todd et al. to identify putative enhancers overlapping with long terminal repeats (roughly 630 elements in embryonic stem cells and 360 in trophoblast stem cells). These elements are called ‘TE+ enhancers’ to distinguish them from traditional ‘TE- enhancers’, which do not share sequences with transposable elements. Most putative TE+ enhancers in embryonic stem cells have already been described, but Todd et al. highlight that these are more specific to certain types of cells than TE- enhancers.

**Figure 1.**
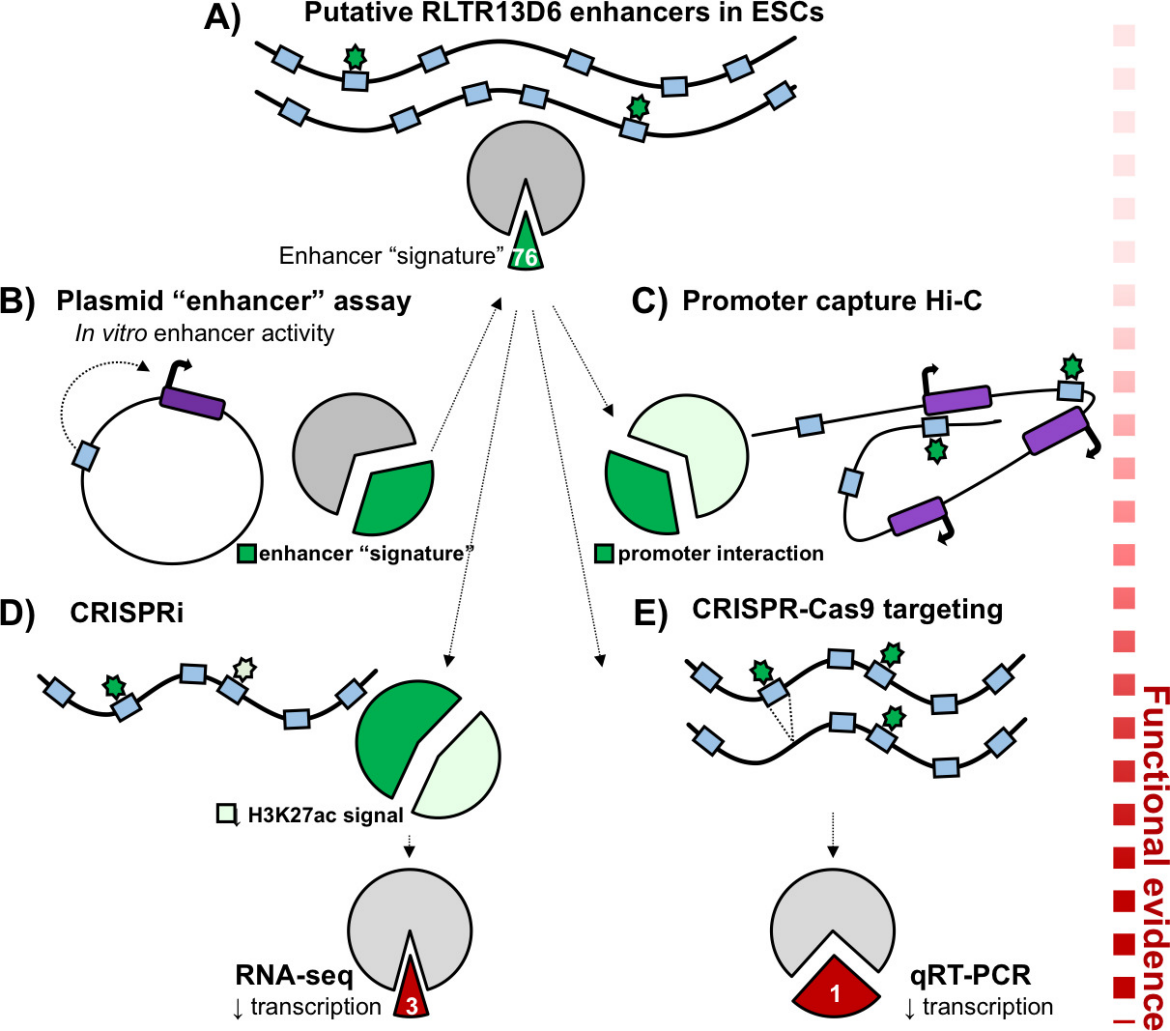
Functional validation of putative TE+ enhancers. Here, the long terminal repeats belonging to the RLTR13D6 family are used to demonstrate how putative enhancers can be validated. (**A**) Of the 805 copies of RLTR13D6 sequences (blue boxes) in the mouse genome, 76 have an ‘enhancer-like’ chromatin state in embryonic stem cells and bind at least one key transcription factor in this cell type (green stars). (**B**) High-throughput plasmid-based reporter assays work by inserting a potential enhancer sequence into a plasmid (black circle), and examining its impact on the expression of a reporter gene (dark purple box). Only a third of the long terminal repeats that show enhancer activity in these assays have an enhancer-like chromatin state in the genome (dark green fraction of the pie chart). (**C**) Promoter capture Hi-C experiments showed that about a third of the putative enhancers (dark green fraction of the pie chart) interacted with the promoter of at least one gene (purple boxes). (**D**) Disrupting RLTR13D6 long terminal repeats using CRISPR interference reduced the histone mark H3K27ac (a sign of enhancer function) by at least two fold for 34 of the 76 sequences (light green). Using RNA-seq after CRISPR interference showed that only three genes associated with an RLTR13D6 element were down-regulated by at least 1.5 fold. (**E**) In embryonic stem cells, CRISPR-Cas9 deletion (blue box disappearing) of four long terminal repeats with enhancer signatures reduced gene expression in only one case.

Plasmid-based reporter assays work by inserting a sequence of interest into a plasmid, and evaluating its impact on the expression of a reporter gene; these experiments have already demonstrated that, in vitro, transposable elements with certain transcription factor binding motifs could play the role of enhancers (Sundaram et al., 2017). Looking at long terminal repeats in which such assays had highlighted a potential enhancer activity, Todd et al. found that, in situ in the genome, only a third of them had chromatin features that were compatible with an enhancer role (Figure 1B). This means that specific sequence features are not enough to predict whether a transposable element works as an enhancer when in the genome: the broader chromatin context in which the element is embedded likely influences whether enhancer-like features can appear.

Since enhancers can act over large distances, Todd et al. took advantage of their previously published promoter chromatin-capture data to identify which genes the putative TE+ enhancers could target. Compared to TE- enhancers, only about 40% of TE+ enhancers were found to physically interact with at least one gene promoter (Figure 1C). These target genes were expressed almost exclusively in embryonic or trophoblast stem cells, which is consistent with the epigenetic profile of TE+ enhancers. In contrast, TE- enhancers tended to interact with genes expressed in a broader range of tissues; this highlights that transposable elements acquire their enhancer-like features in ways that are specific to a cell type.

Finally, Todd et al. harnessed a combination of specific CRISPR-Cas9 deletions and widespread CRISPR interference (Gilbert et al., 2013) to test how TE+ enhancers influenced the expression of the genes they target. The results showed that deleting putative enhancers did not always affect gene expression. In addition, when 76 putative enhancers belonging to the RLTR13D6 family were disrupted in embryonic stem cells, only three of their target genes showed significant reduction in transcription (Figure 1D,E). Chromatin features and exogenous plasmid-based assays can help to map new candidate enhancer regions, but the Branco’s group showcases that, alone, these assays are not enough to confirm enhancer function.

This low validation rate reflects several difficulties that emerge when assessing if sequences with tantalizing epigenomic characteristics are indeed enhancers (discussed in Halfon, 2019). Recent work in humans has demonstrated that primate-specific long terminal repeats are also used as enhancers in human embryonic stem cells (Fuentes et al., 2018; Pontis et al., 2019). Unlike the mouse experiments of Todd et al., the human studies yielded a much higher proportion of putative TE+ enhancers with an impact on gene transcription upon in situ targeting with CRISPR interference. It is not clear whether these differences are due to variations in techniques and significance thresholds, or because humans and mice recruit families of long terminal repeats with enhancer-like roles at a different pace. Nonetheless, this body of work strengthens the theory that transposable elements can act as enhancers, while also highlighting that careful, in situ evaluation is required before any candidate region is given a definite enhancer role.
